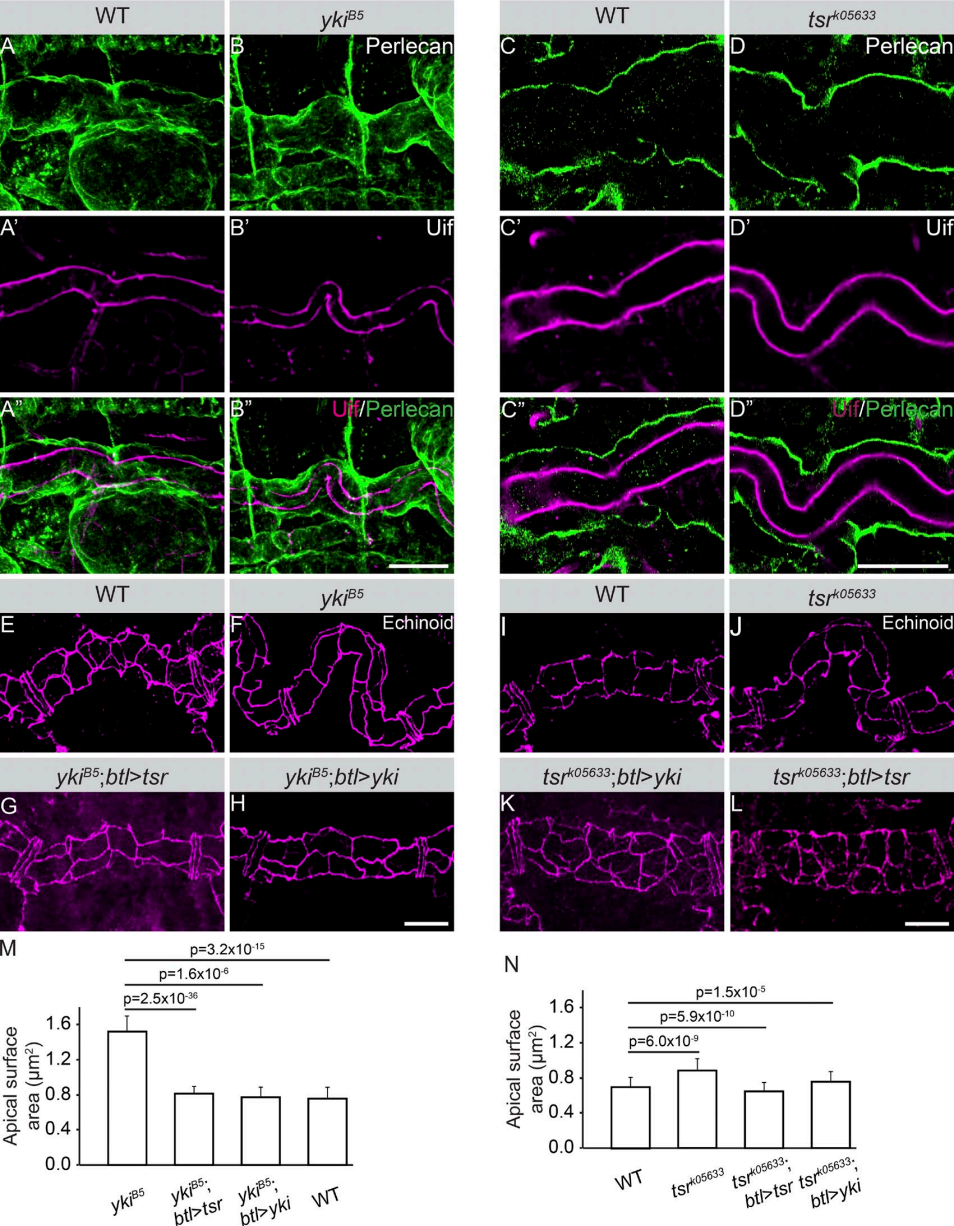# Correction: Yorkie controls tube length and apical barrier integrity during airway development

**DOI:** 10.1083/jcb.20180912104072023c

**Published:** 2023-04-13

**Authors:** Kassiani Skouloudaki, Ioannis Christodoulou, Dilan Khalili, Vasilios Tsarouhas, Christos Samakovlis, Pavel Tomancak, Elisabeth Knust, Dimitrios K. Papadopoulos

Vol. 218, No. 8 | 10.1083/jcb.201809121 | July 17, 2019

After publication, it was discovered that the image in Fig. 8 H showing the rescue of the *yki* mutant convoluted trachea phenotype with a *yki* cDNA from the trachea-specific *btl* enhancer was inadvertently duplicated in Fig. 8 L, which was supposed to show an analogous rescue result for the *tsr* mutant. The corrected figure is shown here. The error appears in print and in PDFs downloaded before April 10, 2023. The authors apologize for the error and any confusion.

**Figure. 8:**